# Predictors of Self-Reported Neglect-like Symptoms and Involuntary Movements in Complex Regional Pain Syndrome Compared to Other Chronic Limb Pain Conditions

**DOI:** 10.1093/pm/pnab226

**Published:** 2021-07-20

**Authors:** Antonia F Ten Brink, Janet H Bultitude

**Affiliations:** 1 Department of Psychology, University of Bath, Bath, UK; 2 Centre for Pain Research, University of Bath, Bath, UK

**Keywords:** Chronic Pain, Complex Regional Pain Syndrome, Neglect-Like Symptoms, Body Perception Disturbance, Involuntary Movements

## Abstract

**Objective:**

In addition to pain, people with complex regional pain syndrome (CRPS) often report inattention to and disengagement from their affected limb (i.e., “neglect-like symptoms”). Understanding how these symptoms relate to other characteristics of CRPS, and chronic pain generally, could provide insights for preventing and treating CRPS.

**Methods:**

We administered an online survey to people who received a diagnosis of CRPS (*n *=* *335) and other chronic limb pain (*n *=* *407). Neglect-like symptoms were assessed using the Neurobehavioral questionnaire.

**Results:**

A principal component analysis identified two components: motor and cognitive neglect-like symptoms, and involuntary movements. Internal consistency of the components was acceptable. We conducted regression analyses with these as outcomes. Having CRPS, a painful lower limb, higher pain intensity, and somatic symptoms were associated with more motor and cognitive neglect-like symptoms. Having CRPS, higher pain intensity, depression, and somatic symptoms were associated with more involuntary movements. Age, gender, anxiety, disease duration, hours of pain per day, affected side, whether the limb was the most painful body part, and number of pain-related medical diagnoses were no predictors. Finally, motor and cognitive neglect-like symptoms were related to tremor; and involuntary movements to changes in skin color, swelling, sweating, toenails, weakness, and tremor.

**Conclusions:**

This study confirms the specificity of inattention to and disengagement from the affected limb in CRPS, independent of other factors. Furthermore, two components of the Neurobehavioral questionnaire were disentangled: motor and cognitive neglect-like symptoms, and involuntary movements. Results could potentially help clinicians to better assess neglect-like symptoms in chronic pain .

## Introduction

Complex regional pain syndrome (CRPS) is a disorder of severe chronic pain in one or more limb(s). Over 75% of people with CRPS report inattention to and disengagement from their affected limb (i.e., “neglect-like symptoms”), such as that their limb does not feel like part of their body, and that they need to focus attention to move it. Such symptoms are also documented in other types of chronic limb pain [1–3] , although typically to a lesser extent [4–6]. Neglect-like symptoms have been related to current pain intensity [4–6], and their extent in acute CRPS predicts higher pain intensity 6 months later [7]. Therefore, they might be a prognostic factor for chronic pain. Understanding their nature and clinical relevance could provide insights into preventing and treating CRPS.

There has been debate on how to refer to neglect-like symptoms in CRPS, as they seem more specific to body perception disturbance than symptoms seen in post-stroke visuospatial neglect [8–12]. Neglect-like symptoms have mainly been assessed using the Neurobehavioral questionnaire created by Galer and Jensen [13], containing two items about cognitive neglect addressing whether the limb feels foreign, two about motor neglect addressing whether directed mental and visual attention is needed to move the limb, and one about involuntary movements (Table 1). Despite this theoretical dissociation, it is unclear whether this questionnaire measures a single, or multiple constructs. If multiple constructs are being measured, their underlying mechanisms might differ, and they may relate differently to clinically relevant outcomes.

**Table 1.** Summary of findings of previous studies on CRPS that used the five-item neurobehavioral questionnaire of Galer and Jensen [13]

Study	CRPS Diagnosis	Means of Assessment	Group	*N*	≥1 Item	Mean or Total Score	1. If I Don’t Focus My Attention … It Would Lie Still, Like Dead Weight	2. … Feels as Though It Is Not Part of My Body	3. I Need to Focus all of My Attention … to Make It Move The Way I Want It To	4. … Sometimes Moves Involuntarily, Without My Control	5. … Feels Dead to Me
Galer and Jensen [13]	Self-report	Dichotomous scale, by mail	CRPS	224	84% (≥1/4)	–	42%	60%	56%	68%	39%
Frettlöh et al. [4]	IASP criteria [14] tested by QST and bone scan	6-point Likert-scale	CRPS	123	90.2%	2.96 ^1^ (95%CI 2.73–3.19)	66.4%	63.3%	76.9%	54.1%	55.7%
			Other limb pain	117	80.3%	2.22^1^ (95%CI 1.98–2.46)	46.9%	53.0%	60.5%	39.1%	46.5%
Reinersmann et al. [1]	Budapest criteria [15] tested by QST and bone scan	6-point Likert-scale	CRPS (upper)	24	70% ^2^	1.7 (SD 1.3)	60% ^2^	65% ^2^	45% ^2^	40% ^2^	45% ^2^
			Other limb pain (upper)	21	80%^2^	1.0 (SD 0.9)	40%^2^	46.6%^2^	26.6%^2^	6.6%^2^	40%^2^
Kolb et al. [2]	Budapest criteria, [15] tested by QST	6-point Likert-scale	CRPS (upper)	20	75%	Tot. 11.55 (SD 1.39)	–	–	–	–	–
			Other limb pain (upper)	20	60%	Tot. 9.95 (SD 1.48)	–	–	–	–	–
Michal et al. [6]	Budapest criteria [15]	6-point Likert-scale	CRPS	50	–	2.4 ^1^ (SD 1.2)	52%	72%	64%	30%	36%
			Other limb pain	27	–	1.9^1^ (SD 1.4)	22.2%	22.2%	33.3%	18.5%	37%
			Migraine	18	–	1.3^1^ (SD 0.5)	16.7%	16.7%	22.2%	5.6%	0%
Wittayer et al. [7]	Budapest criteria [15] tested by QST	6-point Likert-scale	CRPS	53	75%	2.5 (SD 1.39)	–	–	–	–	–
Current study	Self-report	Dichotomous scale, online	CRPS	335	91.6%	2.90 (SD 1.58)	47.8%	64.8%	60.3%	75.8%	41.5%
			Other limb pain	407	68.8%	1.62 (SD 1.59)	27.8%	30.2%	38.3%	44.0%	21.6%

We report on the criteria for the diagnosis of CRPS, means of assessment of the neglect-like symptoms, the number of people per group, the percentage of people who reported at least one neglect-like symptom, the mean or total score of the five items, and the percentage of people who reported neglect-like symptoms for each item. Results for people with CRPS are underlined. CRPS = complex regional pain syndrome; IASP = International Association for the Study of Pain; QST = quantitative sensory testing.

1 The arithmetic mean was computed.

2 The percentages from the study of Reinersmann et al. [1] were provided upon request from the authors, and were not published in the original paper.

Mixed results have been reported regarding the relationship between self-reported neglect-like symptoms as measured with the Neurobehavioral questionnaire and disease characteristics. In people with non-CRPS limb pain, reduced range of motion and joint position sense related to neglect-like symptoms [5]. In people with CRPS, neglect-like symptoms appear unrelated to the ability to use the limb [4, 6]. Most studies, except one [14], found no relationship between neglect-like symptoms and disease duration [4, 6, 7]. Furthermore, there are contradictory findings regarding differences between left and right, and upper and lower limb CRPS [1, 4, 6, 7]. Michal et al. [6] and Wittayer et al. [7] found relationships between neglect-like symptoms and mental distress (e.g., anxiety, depersonalization, somatization, and pain catastrophizing). Aside from these, most studies did not assess independent relationships between neglect-like symptoms, disease characteristics, and mental distress. Therefore, little is known about how neglect-like symptoms relate to these factors. The aims of the current study were to 1) identify the components of the five-item Neurobehavioral questionnaire; 2) assess their internal consistency; 3) compare them between respondents who reported as having received a diagnosis of CRPS and respondents with other chronic limb pain; 4) assess potential predictors (i.e., diagnosis, age, gender, anxiety, depression, somatic symptoms, disease duration, hours of pain per day, pain intensity, affected side, affected extremity, whether the limb was the most painful area, and number of pain-related medical diagnoses); and 5) explore whether they relate to (specific) CRPS symptoms.

## Methods

### Survey

#### Survey Distribution and Demographics

This study formed part of a larger online survey that we created using Qualtrics survey software [15] and distributed between July and December 2018 (see [16] for detailed information). We distributed the survey among people with CRPS who had previously taken part in other studies in our lab, the Community Participant Panel of the Psychology Department of the University of Bath, patient newsletters and social media groups for several pain conditions, and our own social media, friends, and relatives. Information about the study was provided at the start alongside questions pertaining to informed consent.

Respondents were excluded if they gave no informed consent, were aged <16 years, provided double entries, provided inconsistent answers regarding pain duration, did not answer any questions, or had missing data on any of the covariates. Because gender was included as a covariate, we excluded respondents who did not choose male or female as their gender (i.e., the “other” category was too small). Furthermore, for the current study we only included respondents who indicated that they had chronic pain in a limb and who rated one limb as being more painful compared to the others. We did not exclude people with pain in multiple limbs/body areas to obtain a representative sample. Respondents who indicated having received “CRPS” as a diagnosis were allocated to the “CRPS” group, regardless of whether they indicated other pain diagnoses. The other respondents were allocated to the “Other chronic limb pain” group.

The survey took 20–40 minutes to complete. If respondents closed the survey, the answers provided to that point were saved. Respondents had the opportunity to enter a prize draw for one of four £50 Amazon.co.uk vouchers (or a local equivalent). We obtained information about the location of respondents at the moment of filling in the survey where possible. We asked for respondents’ age and gender. The research was approved by the committee on research ethics at the University of Bath (number 18–169), in accordance with the Declaration of the World Medical Association (www.wma.net). Survey questions that were used in the current study are described below and in the Supplementary Data, information on other survey items can be found in Ten Brink et al. [16].

#### Pain Characteristics

We asked whether respondents had experienced pain on most days for ≥3 months, and if so, how long respondents had been experiencing pain, the average hours of pain per day, whether they had received a medical diagnosis for their pain condition, what this diagnosis was, and who they had received their diagnosis from (i.e., which type of medical professional, if any). We predefined 15 pain-related medical diagnoses; including CRPS (we did not dissociate between CRPS I and II, as many people do not know which type they have). An “other” option was included with a free-text box for respondents to specify additional diagnoses; multiple items were counted as separate diagnoses.

Respondents were asked to indicate where in their body they experienced pain over the past week. We measured pain intensity using the Numeric Pain Rating scale [17, 18]. Respondents were asked to select a number on a sliding scale ranging from 0 (“no pain”) to 10 (“worst pain imaginable”) that best reflected the average level of their pain over the last week for each body part that they experienced pain in. We used the pain ratings for the most painful limb for the current study. Respondents were asked what event or injury triggered the onset of their pain condition. We predefined seven events/injuries and included an “other” option with a free-text box.

Two survey questions provided insight into how many pain triggers and bodily changes that reflect symptoms of CRPS [19] were reported. These questions, which we created for the purposes of another study [16], asked which, if any, of 13 pre-defined triggers give the respondent pain; and which of 46 predefined bodily changes respondents have experienced for the first time or that have become worse since the onset of their pain condition. For the current study, we used one item from the pain triggers question (i.e., the touch of clothing/water/breeze), and 13 items from the bodily changes question (i.e., losing hair or extra hair growth, changes in the texture or color of the skin, swelling, changes in the nails of hands/toes, weakness, tremor, sweating more/less, and body parts feeling unusually cold/hot). It is not possible to diagnose CRPS based on these questions, therefore, the sole purpose of this analysis was to provide some insight into CRPS-related characteristics of the two groups.

#### Anxiety and Depression

The Generalized Anxiety Disorder-7 scale (GAD-7) has seven questions with scores ranging from 0 to 3 for each question. Scores indicate mild (5–9), moderate (10–14), or severe (15–21) anxiety [20, 21].

The Patient Health Questionnaire-9 (PHQ-9) has nine questions with scores ranging from 0 to 3 for each question. Scores indicate mild (10–14), moderate (15–19), or severe (20–27) major depression [22, 23].

#### Somatic Symptoms

The Patient Health Questionnaire-15 (PHQ-15) is a standardized and validated measure of somatic symptoms [24]. The PHQ-15 is a 14-item (for men and respondents who choose “other” as their gender) or 15-item (for women) scale for the assessment of somatic symptoms. Respondents answer whether they are “not bothered at all” (0) to “bothered a lot” (2) by specific symptoms such as fainting spells or back pain over the past 4 weeks. Respondents could decline to answer a question about pain or problems during sexual intercourse, in which case this item was scored as 0. Scores represent mild (6–10), moderate (11–15), or severe (15–30) somatic symptoms.

#### Neglect-like Symptoms

Inattention to and disengagement from the most painful limb was measured with the five-item Neurobehavioral questionnaire (Table 2) [13]. Participants choose “true” or “false” for each item, similar to the original version of the questionnaire and consistent with the format of other questions in the survey. In a previous study, the questionnaire that used a Likert-scale showed acceptable to good internal consistency (Cronbach’s alpha CRPS = 0.86; control = 0.77) and could dissociate between people with CRPS and controls [4].

**Table 2.** The five-item Neurobehavioral questionnaire of Galer and Jensen [13] and the concepts that the items are proposed to measure

Item	Proposed Concept
1. If I don't focus my attention on my [painful limb] it would lie still, like dead weight.	Motor neglect
2. My [painful limb] feels as though it is not part of the rest of my body.	Cognitive neglect
3. I need to focus all of my attention on my [painful limb] to make it move the way I want it to.	Motor neglect
4. My [painful limb] sometimes moves involuntarily, without my control.	Involuntary movements
5. My [painful limb] feels dead to me.	Cognitive neglect

#### CRPS Symptoms

To address the question of whether neglect-like symptoms relate to (specific) CRPS symptoms in the current study, we analyzed the 13 items from the predefined bodily changes that reflect symptoms of CRPS [19]: losing hair or extra hair growth, changes in the texture or color of the skin, swelling, changes in the nails of hands/toes, weakness, tremor, sweating more/less, and body parts feeling unusually cold/hot.

### Statistical Analyses

**Figure 1.**
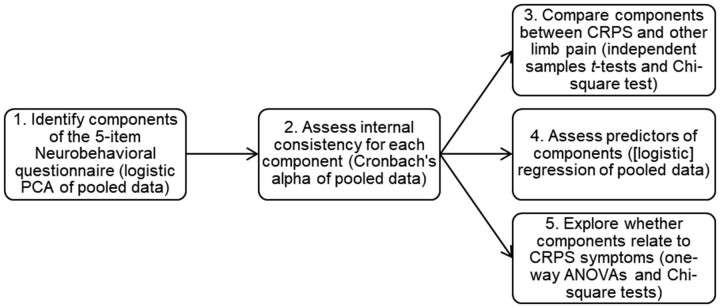
Overview of the statistical analyses on the 5-item Neurobehavioral questionnaire related to five specific sub aims. CRPS = Complex Regional Pain Syndrome; PCA = principal component analysis.

The analysis consisted of five steps corresponding with the five aims of the study (Figure 1). Statistical analysis was conducted using SPSS (version 25). Statistical significance was considered at *P* < .05. Effect sizes were computed with the Pearson correlation coefficient and were considered to reflect a small (>0.10), medium (>0.30), and large effect (>0.50) [25].

#### Demographics, Anxiety, Depression, Somatic Symptoms, and Pain-Related Characteristics

We compared groups regarding age, anxiety, depression, somatic symptoms, pain duration, hours of pain per day, pain intensity, and number of pain-related medical diagnoses using *t*-tests, and regarding gender, most painful limb, whether there was pain in other body areas or other limbs, and which limb was the most painful body area using χ^2^ tests.

#### The Underlying Structure of the Five-Item Neurobehavioral Questionnaire

We performed principal component analysis (PCA) for binary data (logistic PCA) [26] in *R* (version 3.6.0) to explore whether the five questions of the Neurobehavioral questionnaire belong to one component or whether there are more components, such as cognitive neglect-like symptoms (item 2 and 5), motor neglect-like symptoms (item 1 and 3), and involuntary movements (item 4), as suggested in literature [13]. Groups were pooled together for the PCA in order to have larger variability. We repeated the analyses for each group because different mechanisms could be at play in different patient populations. To determine the appropriate number of components, we calculated and plotted the cumulative percent of deviance and the marginal percent of deviance explained by the logistic PCA. We visually inspected where the marginal contributions levelled off. Next, we evaluated how the five items loaded on the different components. We considered items with absolute loadings of above 0.3 as being part of the component.

#### Internal Consistency of the Different Components

For components with more than one item, we computed the internal consistency using the pooled data with Cronbach’s alpha. A Cronbach’s alpha of ≥0.70 was considered acceptable.

#### Differences Between Respondents Who Reported as Having Received a Diagnosis of CRPS versus Other Limb Pain Regarding the Neglect-like Symptoms Components

After determining the components of the questionnaire, we statistically compared uncorrected scores of the components between the two groups, using a *t*-test for the cognitive and motor neglect-like symptoms and a χ^2^ test for the involuntary movements.

#### Predictors of the Neglect-like Symptoms Components

We conducted (ordinal) logistic regression analysis to evaluate which variables predicted the number of symptoms that respondents reported within each component. We used forced entry and included all variables in the model. The dependent variable was the sum score of each component. Potential predictors that we entered in the model were age, gender (male, female), anxiety (GAD-9), depression (PHQ-9), somatic symptoms (PHQ-15), disease duration in years, hours of pain per day, pain intensity of the most painful limb, affected side (left, right), affected extremity (upper, lower), whether the limb was the most painful body area (yes, no), received diagnosis (CRPS, other limb pain), and total number of pain-related medical diagnoses. Entering these variables would not only inform the predictive value of each, but also correct for potential biases based on differences between groups.

#### Relationship Between CRPS Symptoms and the Neglect-like Symptoms Components

To address how the neglect-like components related to CRPS symptoms, for each of the 13 CRPS symptoms we performed one-way ANOVAs for the motor and cognitive neglect-like symptoms and χ2 tests for the involuntary movements, with presence of the symptom as the independent variable (i.e., people who reported as having that symptom versus people who reported as not having that symptom) and the sum score of each component of neglect-like symptoms as the dependent variable. We included all respondents who reported as having received a diagnosis of CRPS. We did not perform a correction for multiple comparisons as these analyses were exploratory.

## Results

### Demographics, Depression, Anxiety, Somatic Symptoms, and Pain-Related Characteristics

Of 2,200 responses, 484 respondents did not give informed consent or closed the survey before answering any question, 13 were aged <16 years, 14 were identified as double entries, 9 gave inconsistent answers, 441 did not have chronic pain, 12 did not choose male or female as being their gender, 242 had missing data on one or more of the covariates due to closing the survey prematurely, and 245 did not report as having pain in a limb or did not report one limb as being more painful than others. This resulted in a sample of 742 respondents, of whom 335 were assigned to the group of respondents who reported as having received a diagnosis of CRPS and 407 to the other limb pain group. We obtained information about the location of 539 respondents (72.6%). Of these respondents, most were located in the United Kingdom (62.0%), the United States (15.8%), Australia (6.7%), the Netherlands (4.3%), Greece (4.3%), Canada (2.0%), Germany (1.9%), and New Zealand (1.9%).

Demographic and pain-related characteristics are depicted in Table 3. Groups were comparable in the distribution of age and gender. Respondents who reported as having received a diagnosis of CRPS obtained higher scores for anxiety, depression, and pain intensity; and reported more hours of pain per day compared to respondents with other limb pain, which were small effects. Respondents with other limb pain reported more somatic symptoms and a longer pain duration than respondents who reported as having received a diagnosis of CRPS, with small effect sizes. In both groups, the lower limb was more often affected than the upper limb. In respondents who reported as having received a diagnosis of CRPS, the left limb was more often affected than in respondents with other limb pain. More respondents with other limb pain reported that they had pain in other parts of their body in addition to the painful limb compared to respondents who reported as having received a diagnosis of CRPS. More respondents who reported as having received a diagnosis of CRPS reported that one of their limbs was the most painful body part compared to the other limb pain group. In the group of respondents who reported as having received a diagnosis of CRPS, 91.9% reported at least one symptom in three or more categories, which was 37.8% in the other limb pain group (Supplementary Data).

**Table 3.** Demographics, depression, anxiety, somatic symptoms, and pain-related characteristics; means (SD) and frequencies (%), split for the respondents who reported as having received a diagnosis of CRPS (“CRPS”) and the respondents who had chronic pain but did not report as having received a diagnosis of CRPS (“other limb pain”)

	CRPS	Other Limb Pain	*t*-Test or χ^2^ Test Statistics
	(*N *=* *335)	(*N *=* *407)	
Age, in years	46.64 (12.08)	46.36 (13.65)	*t*(736.08) = 0.29, *P* = .771, *r* = 0.01
Gender, % female	297 (88.7%)	363 (89.2%)	χ^2^(1) = 0.05, *P* = .818
Anxiety (GAD-7; 0–21)	10.71 (5.95)	9.80 (5.98)	*t*(740) = 2.09, *P* = .037, *r* = 0.08
Depression (PHQ-9; 0–27)	15.87 (6.39)	14.46 (6.77)	*t*(740) = 2.91, *P* = .004, *r* = 0.11
Somatic symptoms (PHQ-15; 0–30)	13.74 (4.98)	14.65 (5.43)	*t*(740) = −2.36, *P* = .019, *r* = 0.09
Pain-related characteristics			
Pain duration in years	8.80 (8.32)	12.28 (10.71)	*t*(737.68) = −4.98, *P* < .001, *r* = 0.18
Hours of pain per day	18.10 (6.99)	14.15 (7.87)	*t*(735.64) = 7.24, *P* < .001, *r* = 0.26
Pain intensity most painful limb (0–10)	7.41 (1.97)	6.48 (2.06)	*t*(740) = 6.27, *P* < .001, *r* = 0.22
Most painful limb, % upper	118 (35.2%)	149 (36.6%)	χ^2^(1) = 0.15, *P* = .696
Most painful limb, % left	158 (47.2%)	156 (38.3%)	χ^2^(1) = 5.88, *P* = .015
Pain in other body areas, % yes	250 (74.6%)	393 (96.6%)	χ^2^(1) = 76.46, *P* < .001
Pain in other limbs, % yes	163 (48.7%)	320 (78.6%)	χ^2^(1) = 72.63, *P* < .001
The limb is (one of) the most painful body area(s), % yes	282 (84.2%)	164 (40.3%)	χ^2^(1) = 147.58, *P* < .001
Number of pain-related medical diagnoses	2.47 (1.88)	2.93 (2.03)	*t*(740) = −3.19, *P* = .001, *r* = 0.12

CRPS = complex regional pain syndrome; GAD-7 = Generalized Anxiety Disorder-7; PHQ-9 = Patient Health Questionnaire-9; PHQ-15 = Patient Health Questionnaire-15.

Respondents with other limb pain reported a higher number of pain-related medical diagnoses than respondents who reported as having received a diagnosis of CRPS, which was a small effect. The pain-related medical diagnoses are depicted in Table 4. In Supplementary Data, we show which medical practitioner(s) (if any) respondents reported as having provided the medical diagnosis. Information on events/injuries that triggered the pain condition are also presented in Supplementary Data.

**Table 4.** Numbers and percentages of pain-related medical diagnoses split for the respondents who reported as having received a diagnosis of CRPS (“CRPS”) and the respondents who had chronic pain but did not report as having received a diagnosis of CRPS (“other limb pain”)

	CRPS	Other Limb Pain	χ^2^ Test Statistics
	(*N *=* *335)	(*N *=* *407)	
CRPS	335 (100%)	0	–
Back pain	88 (26.3%)	147 (36.1%)	χ^2^(1) = 8.24, *P* = .004
Fibromyalgia	61 (18.2%)	263 (64.6%)	χ^2^(1) = 160.90, *P* < .001
Osteoarthritis	51 (15.2%)	106 (26.0%)	χ^2^(1) = 12.90, *P* < .001
Migraine	46 (13.7%)	101 (24.8%)	χ^2^(1) = 14.21, *P* < .001
Irritable bowel disease	40 (11.9%)	123 (30.2%)	χ^2^(1) = 35.82, *P* < .001
Hypermobility condition	27 (8.1%)	71 (17.4%)	χ^2^(1) = 14.12, *P* < .001
Neuralgia	25 (7.5%)	30 (7.4%)	χ^2^(1) = 0.002, *P* = .962
Plantar fasciitis	20 (6.0%)	41 (10.1%)	χ^2^(1) = 4.10, *P* = .043
Rheumatoid arthritis	17 (5.1%)	36 (8.8%)	χ^2^(1) = 3.94, *P* = .047
Endometriosis	12 (3.6%)	17 (4.2%)	χ^2^(1) = 0.17, *P* = .677
Cluster headache	7 (2.1%)	15 (3.7%)	χ^2^(1) = 1.63, *P* = .202
Stomach ulcer	5 (1.5%)	8 (2.0%)	χ^2^(1) = 0.24, *P* = .625
Crohn’s Disease	1 (0.3%)	2 (0.5%)	χ^2^(1) = 0.17, *P* =.680
Multiple Sclerosis	2 (0.6%)	1 (0.2%)	χ^2^(1) = 0.56, *P* = .453
Other (one or more other pain-related diagnosis)	58 (17.3%)	154 (37.8%)	χ^2^(1) =37.93, *P* < .001
None	0	32 (7.9%)	–

Note that respondents could report multiple diagnoses, thus percentages do not sum to 100. CRPS = complex regional pain syndrome.

### The Underlying Structure of the Five-Item Neurobehavioral Questionnaire

**Figure 2.**
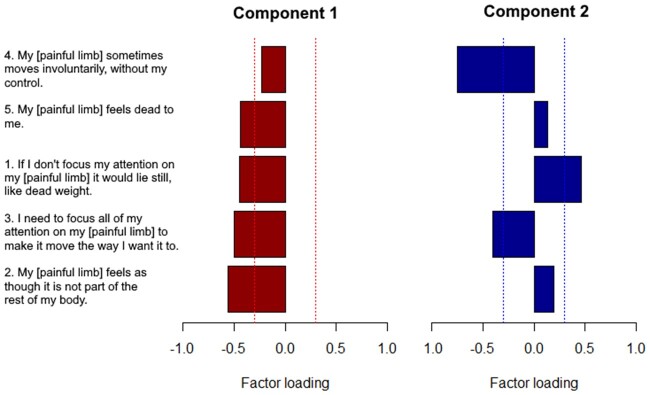
Factor loadings of the five items for the two components, sorted by the factor loadings on the first component for ease of interpretation. Reference lines are depicted at −0.3 and 0.3, which we used as the threshold for considering items as being part of a component. Note that the direction of the factor loading (positive or negative) only has meaning in comparison with the other factor loadings.

Based on visual inspection of the marginal percent of deviance explained, two components were retained. Here, we report the component characteristics based on the PCA that included all respondents. Figure 2 depicts a graphical representation of the factor loadings on each component. Similar components were retained when we analyzed the CRPS and other limb pain groups separately (Supplementary Data).

The first and the second component explained 46% and 21% of the variance, respectively. The four items that loaded on the first component where item 2 (−0.56), item 3 (−0.50), item 1 (−0.45), and item 5 (−0.44). This component reflects the motor and cognitive neglect-like symptoms described in the literature. Item 4 was, with a factor loading of −0.23, no part of the first component. The three items that loaded on the second component were item 4 (−0.75), item 3 (−0.40), and item 1 (0.46). Note that people who tend to *agree* with item 4 and item 3, and *disagree* with item 1, or the other way around, would obtain high scores for this component. As the items are not supposed to be recoded, we did not include item 1 in the second component. Because item 3 had a higher factor loading on the first than the second component, and based on previous literature, we decided to retain component 1, “motor and cognitive neglect-like symptoms” (including item 1, 2, 3, and 5), and separately analyze item 4 “involuntary movements,” so that none of the items would overlap between the components.

### Internal Consistency of the Different Components

The internal consistency of the “motor and cognitive neglect-like symptoms” component was acceptable (Cronbach’s alpha = 0.76). As the involuntary movement component only consisted of one item, its internal consistency was not evaluated.

### Differences Between Respondents Who Reported as Having Received a Diagnosis of CRPS versus Other Limb Pain regarding the Neglect-like Symptoms Components

The two groups differed regarding the first component; the number of motor and cognitive neglect-like symptoms, *t*(740)=10.98, *P* < .001, *r *=* *0.37. Respondents who reported as having received a diagnosis of CRPS reported more motor and cognitive neglect-like symptoms (*M* = 2.14, *SD *=* *1.42) than respondents with other limb pain (*M* = 1.18, SD* *=* *1.39), which was a moderate effect. Groups also differed regarding the second component; the percentage of respondents who reported involuntary movements, χ^2^(1)=76.66, *P <* .001. Respondents who reported as having received a diagnosis of CRPS more often reported involuntary movements (75.8%) than respondents with other limb pain (44.0%). According to the odds ratio, respondents who reported as having received a diagnosis of CRPS were four times more likely to report involuntary movements than respondents with other limb pain.

### Predictors of the Neglect-like Symptoms Components

#### Predictors of the Motor and Cognitive Neglect-like Symptoms

The ordinal regression model was significant (*P <* .001) and the model explained 22.2% (Nagelkerke *R*^2^) of the variance in motor and cognitive neglect-like symptoms (Table 5). The odds of respondents who reported as having received a diagnosis of CRPS reporting motor and cognitive neglect-like symptoms was 3.07 times that of respondents with other limb pain, Wald χ2(1)=47.73, *P <* .001. Higher overall pain intensity was associated with higher odds of reporting motor and cognitive neglect-like symptoms, Wald χ^2^(1)=8.22, *P =* .004. A higher level of somatic symptoms was associated with higher odds of reporting motor and cognitive neglect-like symptoms, Wald χ^2^(1)=7.69, *P =* .006. The odds of respondents with a painful lower limb to report motor and cognitive neglect-like symptoms was 1.40 times of that of respondents with a painful upper limb, Wald χ^2^(1) =5.48, *P =* .019.

**Table 5.** Ordinal regression results predicting motor and cognitive neglect-like symptoms (ranging from 0 to 4), including respondents who reported as having received a diagnosis of CRPS (“CRPS”; *N *=* *335) and the respondents who had chronic pain but did not report as having received a diagnosis of CRPS (“other limb pain”; *N *=* *407)

	B	SE	OR (95% CI)	*P*
Age, in years	−0.001	0.005	1.00 (0.99 to 1.01)	.922
Pain duration in years	−0.006	0.008	0.99 (0.98 to 1.01)	.419
Hours of pain per day	−0.013	0.010	0.99 (0.97 to 1.01)	.193
Pain intensity of the most painful limb	0.121	0.042	1.13 (1.04 to 1.23)	.004*
Number of medical diagnoses	−0.069	0.039	0.93 (0.86 to 1.01)	.080
Anxiety (GAD-7)	0.019	0.018	1.02 (0.98 to 1.06)	.287
Depression (PHQ-9)	0.035	0.018	1.04 (1.00 to 1.07)	.052
Somatic symptoms (PHQ-15)	0.051	0.018	1.05 (1.02 to 1.09)	.006*
Group = CRPS (vs other limb pain)	1.121	0.162	3.07 (2.23 to 4.21)	< .001**
Gender = male (vs female)	0.093	0.221	1.10 (0.71 to 1.69)	.672
Limb side = left (vs right)	0.025	0.137	1.03 (0.78 to 1.34)	.855
Limb extremity = lower (vs upper)	0.333	0.142	1.40 (1.06 to 1.84)	.019*
Limb is the most painful body area = no (vs yes)	−0.154	0.167	0.86 (0.62 to 1.19)	.355

CRPS = complex regional pain syndrome; GAD-7 = Generalized Anxiety Disorder-7; OR = adjusted odds ratio; PHQ-9 = Patient Health Questionnaire-9; PHQ-15 = Patient Health Questionnaire-15; SE = standard error.

Asterisks indicate statistical significance with alpha < 0.05*, and with alpha < 0.001**.

#### Predictors of the Involuntary Movements

The logistic regression model was significant, χ^2^(13)=169.60, *P <* .001. The model explained 27.5% (Nagelkerke *R*^2^) of the variance in involuntary movements and correctly classified 70.4% of cases (Table 6). Respondents who reported as having received a diagnosis of CRPS were 4.55 times more likely to report involuntary movements than respondents with other limb pain, Wald χ^2^(1)=54.06, *P <* .001. Higher levels of pain intensity, Wald χ^2^(1)=6.55, *P =* .010, depression, Wald χ^2^(1)=6.53, *P =* .011, and somatic symptoms, Wald χ^2^(1)=10.48, *P =* .001, were associated with an increased likelihood of reporting involuntary movements.

**Table 6.** Logistic regression results predicting involuntary movements (present versus absent), including respondents who reported as having received a diagnosis of CRPS (“CRPS”; *N *=* *335) and the respondents who had chronic pain but did not report as having received a diagnosis of CRPS (“other limb pain”; *N *=* *407)

	B	SE	OR (95% CI)	*P*
Age, in years	−0.009	0.007	0.99 (0.98 to 1.00)	.181
Pain duration in years	0.010	0.009	1.01 (0.99 to 1.03)	.282
Hours of pain per day	0.016	0.012	1.02 (0.99 to 1.04)	.214
Pain intensity of the most painful limb	0.133	0.052	1.14 (1.03 to 1.26)	.010*
Number of medical diagnoses	−0.050	0.049	0.95 (0.86 to 1.05)	.287
Anxiety (GAD-7)	−0.040	0.023	0.96 (0.92 to 1.01)	.082
Depression (PHQ-9)	0.058	0.023	1.06 (1.01 to 1.11)	.011*
Somatic symptoms (PHQ-15)	0.076	0.023	1.08 (1.03 to 1.13)	.001*
Group = CRPS (vs other limb pain)	1.514	0.206	4.55 (3.04 to 6.81)	< .001**
Gender = male (vs female)	−0.299	0.280	0.74 (0.43 to 1.28)	.285
Limb side = left (vs right)	−0.152	0.172	0.86 (0.61 to 1.20)	.377
Limb extremity = lower (vs upper)	0.203	0.175	1.23 (0.87 to 1.73)	.245
Limb is the most painful body area = no (vs yes)	0.192	0.206	1.21 (0.81 to 1.81)	.352

CRPS = complex regional pain syndrome; GAD-7 = Generalized Anxiety Disorder-7; OR = adjusted odds ratio; PHQ-9 = Patient Health Questionnaire-9; PHQ-15 = Patient Health Questionnaire-15; SE = standard error.

Asterisks indicate statistical significance with alpha < 0.05*, and with alpha < 0.001**.

### Relationship Between CRPS Symptoms and Neglect-like Symptoms Components

#### Relationship Between CRPS Symptoms and the Motor and Cognitive Neglect-like Symptoms

Respondents who reported as having experienced changes in their toenails, and tremor in any part of their body, reported more motor and cognitive neglect-like symptoms compared to respondents without these symptoms, which were small effects (Table 7).

**Table 7.** Average number of motor and cognitive neglect-like symptoms (ranging from 0 to 4) given by respondents who reported as having received a diagnosis of CRPS (*N *=* *335)

	Respondents with Symptom			Respondents Without Symptom			One-Way ANOVA Statistics
	*N*	Mean (SD)	*N*		Mean (SD)		
Losing hair on parts of your body other than your head	62	2.26 (1.58)	273		2.12 (1.39)	*F*(1) = 0.50, *P* = .482, *r* = 0.04	
Extra hair growth on any part of your body	115	2.25 (1.33)	220		2.09 (1.47)	*F*(1) = 1.03, *P* = .312, *r* =0.06	
Changes in the texture of your skin	214	2.19 (1.45)	121		2.06 (1.37)	*F*(1) = 0.68, *P* = .409, *r* = 0.05	
Changes in skin color	259	2.21 (1.44)	76		1.91 (1.34)	*F*(1) = 2.71, *P* = .101, *r* = 0.09	
Swelling (edema) in any body part	277	2.19 (1.43)	58		1.90 (1.35)	*F*(1) = 2.12, *P* = .146, *r* = 0.08	
Changes in the nails of your hands (e.g., growing faster or slower, or being more brittle)	180	2.17 (1.46)	155		2.12 (1.39)	*F*(1) = 0.11, *P* = .746, *r* = 0.02	
Changes in your toenails (e.g., growing faster or slower, or being more brittle)	189	2.30 (1.40)	146		1.94 (1.43)	*F*(1) = 5.45, *P* = .020*, *r* = 0.13	
Changes in nails of hands **or** toes	254	2.22 (1.41)	81		1.89 (1.45)	*F*(1) = 3.45, *P* = .064, *r* = 0.10	
Weakness in any part of your body	285	2.21 (1.43)	50		1.78 (1.33)	*F*(1) = 3.87, *P* = .050, *r* =0.11	
Tremor in any part of your body	191	2.34 (1.39)	144		1.88 (1.42)	*F*(1) = 8.73, *P* = .003*, *r* = 0.16	
Sweating more	223	2.24 (1.39)	112		1.95 (1.46)	*F*(1) = 3.25, *P* = .072, *r* = 0.05	
Sweating less	14	2.21 (1.81)	321		2.14 (1.41)	*F*(1) = 0.04, *P* = .849, *r* = 0.01	
One part or specific parts of your body feeling unusually cold	207	2.13 (1.43)	128		2.17 (1.42)	*F*(1) = 0.08, *P* = .773, *r* = 0.02	
One part or specific parts of your body feeling unusually hot	142	2.17 (1.40)	193		2.12 (1.44)	*F*(1) = 0.08, *P* = .777, *r* = 0.02	

CRPS = complex regional pain syndrome. Asterisks indicate statistical significance with alpha < 0.05*, and with alpha < 0.001**.

Note. Data are split and compared according to whether or not respondents also reported CRPS symptoms. Even though CRPS symptoms can spread across (ipsilateral) limbs [28–32], reporting changes in toenails could reflect having CRPS in a lower limb versus CRPS in an upper limb, instead of being specific for experiencing changes in nails. The variable “Changes in nails of hands **or** toes” was created based on the two variables asking about changes in the nails of the hands and toenails. Respondents who gave a positive answer to either one of those two variables were categorized as respondents with the symptom. Indeed, of people who reported an upper limb as being most painful (*n = *118), 70.3% reported changes in the nails of their hands and 33.1% in their toenails. Of people who reported a lower limb as being most painful (*n *=* *217), 44.7% reported changes in the nails of their hands and 79.5% in their toenails. Differences between groups were significant (both *P <* .001). Therefore, we created an additional variable based on the questions “Changes in the nails of your hands” and “Changes in your toenails.” Respondents who gave a positive answer to either one of those questions were categorized as respondents with “Changes in nails on hands **or** toes.” There was no difference in the number of motor and cognitive neglect-like symptoms between respondents who reported changes in nails on hands or toes versus respondents who did not report such changes. This indicates that reporting changes in toenails, rather than nails per se, was specifically related to neglect-like symptoms, suggesting that this was driven by lower limb CRPS rather than changes in nails.

#### Relationship Between CRPS Symptoms and the Involuntary Movements

Respondents who reported as having experienced changes in skin color, swelling, toenails, nails of hands or toes, weakness, tremor, or sweating more; reported experiencing involuntary movements of their most painful limb more often compared to respondents without these symptoms (Table 8).

**Table 8.** Percentages of respondents who reported as having received a diagnosis of CRPS (*N *=* *335), who reported involuntary movements

	Respondents with Symptom		Respondents Without Symptom		χ^2^ Test Statistics
	*N*	% reporting involuntarymovements	*N*	% reporting involuntarymovements	
Losing hair on parts of your body other than your head	62	82.3%	273	74.4%	χ^2^(1) =1.72, *P* = .190
Extra hair growth on any part of your body	115	79.1%	220	74.1%	χ^2^(1) = 1.05, *P* = .306
Changes in the texture of your skin	214	78.0%	121	71.9%	χ^2^(1) = 1.59, *P* = .208
Changes in skin color	259	78.4%	76	67.1%	χ^2^(1) = 4.07, *P* = .044*
Swelling (edema) in any body part	277	78.0%	58	65.5%	χ^2^(1) = 4.06, *P* = .044*
Changes in the nails of your hands (e.g., growing faster or slower, or being more brittle)	180	79.4%	155	71.6%	χ^2^(1) = 2.79, *P* = .095
Changes in your toenails (e.g., growing faster or slower, or being more brittle)	189	84.1%	146	65.1%	χ^2^(1) = 16.32, *P* < .001**
Changes in nails of hands **or** toes	254	80.3%	81	61.7%	χ^2^(1) = 11.57, *P* = .001*
Weakness in any part of your body	285	79.3%	50	56.0%	χ^2^(1) = 12.60, *P* < .001**
Tremor in any part of your body	191	85.9%	144	62.5%	χ^2^(1) = 24.45, *P <* .001**
Sweating more	223	80.3%	112	67.0%	χ^2^(1) = 7.20, *P* = .007*
Sweating less	14	85.7%	321	75.4%	χ^2^(1) = 0.78, *P* = .377
One part or specific parts of your body feeling unusually cold	207	77.8%	128	72.7%	χ^2^(1) = 1.13, *P* = .287
One part or specific parts of your body feeling unusually hot	142	80.3%	193	72.5%	χ^2^(1) = 2.68, *P* = .102

CRPS = complex regional pain syndrome. Asterisks indicate statistical significance with alpha < 0.05*, and with alpha < 0.001**.

Note. Data are split and compared according to whether or not respondents also reported CRPS symptoms. The variable “Changes in nails of hands **or** toes” was created based on the two variables asking about changes in the nails of the hands and toenails. Respondents who gave a positive answer to either one of those two variables were categorized as respondents with the symptom.

## Discussion

We evaluated the underlying structure of the five-item Neurobehavioral questionnaire of Galer and Jensen [13], and examined how different components related to demographic and clinical characteristic, and specific CRPS symptoms. Motor and cognitive neglect-like symptoms clustered together, the item on involuntary movements was a separate component. On average, both respondents who reported as having received a diagnosis of CRPS and those with other chronic limb pain reported at least one of the five symptoms, showing that these are not exclusive to CRPS. However, people who reported as having received a diagnosis of CRPS reported more motor and cognitive neglect-like symptoms, and involuntary movements, than people with other limb pain conditions, when controlled for age, gender, anxiety, depression, somatic symptoms, disease duration, hours of pain per day, pain intensity, affected side, affected extremity, whether the limb was the most painful, and number of pain-related medical diagnoses.

The clustering of the motor and cognitive neglect-like symptoms indicates that they relate to a similar underlying mechanism, which is different from the mechanism underlying involuntary movements. It should be stressed that motor and cognitive neglect-like symptoms in CRPS differ from hemispatial neglect after stroke [8–10], which most often manifests as a visuospatial bias. Typically, people with CRPS perform normally on classic “pen-and-paper” neglect tasks (e.g., line bisection), which capture a combination of perceptual and motor biases that stroke patients generally are not aware of ([1, 2, 14, 27, 28], although see [29, 30]), and people with CRPS show no visuospatial attention bias towards one side of space ([31, 32], although see [33, 34]). Motor and cognitive neglect-like symptoms in CRPS more closely resemble a less common manifestation of post-stroke neglect called “personal neglect” (e.g., failure to dress or groom the contralesional side of the body), which might be primarily a disorder of body perception rather than attention [35]. Therefore, the Neurobehavioral questionnaire should be considered a measure of body perception disturbances and could be complemented with, for example, the Bath CRPS Body Perception Disturbance scale, which together provide a more complete picture of body perception disturbances [12, 36]. The origin of such body perception disturbances in CRPS remains unclear. Galer et al. [37] suggested that it could reflect dysfunction in central nervous system structures, in particular cortical reorganization of parietal cortex function [7]. In parietal areas, input from sensory systems is integrated and form the body image, and lesions in parietal areas are related to post-stroke (motor) neglect [38, 39]. The degree of cortical reorganization correlates with pain severity and body perception disturbances [40–43]. This suggests a relationship between altered cortical limb representation, pain intensity, and disturbances in limb perception [8]. Possibly, the motor neglect-like symptoms directly stem from the cognitive neglect-like symptoms causing them to cluster together: people need to feel like their limb is part of their body in order to feel that they can easily move their own limb, or vice versa.

There are several potential mechanisms of movement disorders in chronic pain, acting at different levels of the sensorimotor circuitry [44]. For example, nociceptive neurons in the spinal cord may become sensitized. Pain becomes chronic and normally non-painful stimuli become painful (central sensitization). Central sensitization might influence the spinal motor circuitry, leading to loss of voluntary control and movement disorders [45, 46].

Both components of the Neurobehavioral questionnaire were related to having received a diagnosis of CRPS, more intense pain, and more somatic symptoms, consistent with previous research [4–7]. However, each component also had unique predictors. Motor and cognitive neglect-like symptoms were predicted by having a lower painful limb opposed to having an upper painful limb. Previous smaller studies (*n *≤* *20) found either more [4], a similar number of [6], or fewer [7] neglect-like symptoms in people with lower versus upper limb CRPS. Our larger sample size allowed us to control for several potentially confounding variables. A lower limb dominance is also seen in xenomelia, where people feel as if a body part does not belong to them, and experience a desire to amputate, paralyze, or disable it [47, 48]. The lower limb dominance of xenomelia, which could also explain the neglect-like symptoms in respondents who reported as having received a diagnosis of CRPS, has been explained by involvement of the vestibular system, which contributes to maintaining a coherent body representation [49–51] and principally receives input from the lower limbs [52, 53]. Another explanation is that the insula, a core region in xenomelia as it is associated with the integration of body and mind, is anatomically close to the leg representation on the secondary somatosensory cortex, and, therefore, particularly important in lower limb representation [48]. Involuntary movements were predicted by depression, and a similar trend was seen for the motor and cognitive neglect-like symptoms. This is in line with the only other study in which the relationship between neglect-like symptoms (all five items) and depression was measured [6]. The authors concluded that mental distress might contribute to the development of neglect-like symptoms, especially through depersonalization and catastrophizing. However, the direction of the relationship is unknown: it might as well be that involuntary movements, and possibly motor and cognitive neglect-like symptoms, lead to mental distress.

Finally, while both components were predicted by general somatic symptoms (e.g., dizziness, feeling tired), exploratory analyses showed that involuntary movements were related to a greater number of CRPS-specific symptoms across all four diagnostic categories (vasomotor, sudomotor/edema, trophic, and motor). To some extent, involuntary movements are part of the diagnostic criteria for CRPS in the form of tremor and dystonia [19]. This suggests that the mechanism(s) underlying this component might be more closely linked with those that results in physical CRPS symptoms, whereas less related mechanism(s) might underly motor and cognitive neglect-like symptoms. These analyses were exploratory and warrant further research.

### Limitations and Strengths

This study has some limitations. First, to maximize sample size we conducted an online survey in order to include people who live distant from our lab and/or are not able to travel. Groupings were, therefore, based on self-reported diagnoses. To mitigate this, we asked respondents to report from whom they received their diagnoses. Most respondents reported receiving their diagnoses from an appropriately qualified practitioner. Furthermore, our analyses of clinical characteristics are consistent with previous research. Respondents who reported as having received a diagnosis of CRPS reported higher levels of anxiety and depression than respondents with other pain [54], their pain onset was mostly associated with physical trauma, and they most frequently reported their limb(s) as being the most painful body part. In addition, 91.9% of respondents who reported as having received a diagnosis of CRPS reported at least one CRPS-related symptom in three or more categories, compared to 37.8% for the other limb pain group. Importantly, these numbers do not reflect a CRPS diagnosis, and we did not assess all CRPS symptoms in our questionnaire (i.e., not hyperesthesia, decreased range of movement, dystonia). Therefore, it is possible that some respondents who reported as having received a diagnosis of CRPS did not fulfill the CRPS criteria, whereas some respondents in the other limb pain group did, but never had received any diagnosis. Furthermore, we cannot draw conclusions on the relationships between all CRPS symptoms and neglect-like symptoms. Nevertheless, even with this crude group categorization moderate differences between groups were observed. A second limitation is that we did not dissociate between CRPS type I and II. As neglect-like symptoms are often attributed to central mechanisms, and neuronal damage could be related to such mechanisms, differences between these subtypes possibly exist. Third, to obtain representative samples, we did not limit our inclusion to people with unilateral pain. Since having pain in more than one limb could have affected neglect-like symptoms, this was included as a covariate in our regression analyses. Fourth, we recorded only true/false responses for the Neurobehavioral questionnaire, rather than using an alternative version that asks participants to rate the extent of each symptom on a 6-item scale [4]. We were therefore unable to make inferences about the severity or frequency of these symptoms, and it could have reduced sensitivity of the regression models. It has, however, been shown that the number of neglect-like symptoms is specific for CRPS versus general chronic limb pain, as, for example, more people with CRPS confirm all five items than people with other pain [4], which we indeed found. Nevertheless, the internal consistency in the current study was lower compared to the study using the Likert scale. Therefore, we recommend using a Likert scale in future studies. Finally, the internal consistency should be further verified in a group with confirmed CRPS.

Strengths are that we included a larger sample of people compared to other studies, enabling us to control for several potentially confounding factors. Second, we evaluated the relationship between neglect-like symptoms and depression, anxiety, number of medical diagnoses, and hours of pain per day, which has not been investigated before. We evaluated the independent relationships of these and other variables instead of looking at those variables in isolation. This is crucial, as some of these variables are both related to a specific diagnosis and to an increased likelihood of reporting neglect-like symptoms. Finally, we were the first to explore the relationships between neglect-like symptoms and CRPS symptoms.

## Conclusion

The five-item Neurobehavioral questionnaire of Galer and Jensen [13] measures two components: motor and cognitive neglect-like symptoms, and involuntary movements. Their internal consistency was acceptable. Both components are reported more frequently by people who reported as having received a diagnosis of CRPS as opposed to people with other chronic limb pain, and are associated with higher pain intensity and more somatic symptoms. The motor and cognitive neglect-like symptoms were more related to lower versus upper limb pain, whereas the involuntary movements related more to depression. Finally, our results confirm previous findings on relationships between neglect-like symptoms and clinically relevant outcomes, and stress the importance of assessing body perception disturbances in clinical practice. Dissociating between the two components in future studies is relevant as they might reflect different mechanisms which could be differently related to clinical outcomes.

## Author Contributions

***Antonia Ten Brink***: Conceptualization, Investigation, Formal analysis, Writing—Original Draft, Funding acquisition. ***Janet Bultitude***: Conceptualization, Methodology, Software, Resources, Writing—Review and Editing, Supervision, Funding acquisition.

## Supplementary Material

pnab226_Supplementary_Data

## Notes

Funding source: Antonia ten Brink was supported by a Rubicon grant (019.173SG.019) from the Netherlands Organisation for Scientific Research (NWO). The funder had not role in study design, analysis and interpretation of data, writing the report, and the decision to submit the article for publication.

Conflict of interest/Disclosure summary: There are no conflicts of interest to report. These results have not been published in any form before. Antonia ten Brink was supported by a Rubicon grant (019.173SG.019) from the Netherlands Organisation for Scientific Research (NWO). The funder had not role in study design, analysis and interpretation of data, writing the report, and the decision to submit the article for publication.
