# Supplementary material for: Predictors of Self-Reported Neglect-like Symptoms and Involuntary Movements in Complex Regional Pain Syndrome Compared to Other Chronic Limb Pain Conditions
Source: Pain Med. 2021 Jul 20;22(10):2337–49. doi: 10.1093/pm/pnab226 (PMC8664463; doi:10.1093/pm/pnab226)
Supplement: pnab226_Supplementary_Data [file pnab226_supplementary_data.pdf]

Survey questions

Demographic and pain-related information

What is your age in years?

▼ Under 16 ... 100 or older

What is your gender?

▼ Male, Female, Other

Have you been experiencing pain on most days for three months or more?

- Yes
- No

On average, for how many **hours** per day do you normally feel pain? Please answer using **numbers**. For example, half an hour would be ".5", and two hours would be "2".

Hours per day ____

For approximately how long have you been experiencing pain? Please answer in **years** and **months**. For example, 6 months would be “0" years and "6" months.

Years ____

Months ____

Where in your body have you felt pain **over the last week**. You can select as many responses as you like so please select all that apply.

- Left arm and/or hand
- Left leg and/or foot
- Right arm and/or hand
- Right leg and/or foot
- Back
- Stomach/abdomen
- Chest
- Groin/genitals
- Neck
- Head
- Other (Please specify. You can specify more than one thing if you wish)

For each of the body parts that you have selected, please rate your **average** level of pain that you have experienced in that body part **over the last week**.

No pain Worst pain imaginable

0 1 2 3 4 5 6 7 8 9 10

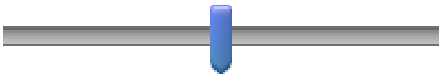

Have you received a medical diagnosis for your pain condition? Here, a medical diagnosis is a diagnosis that has been determined by a medical practitioner such as a GP, specialist doctor (e.g. rheumatologist or pain specialist), physiotherapist, occupational therapist, or nurse.

- o Yes
- o No

Please select all medical diagnoses that you have received for your pain condition.

- o Complex Regional Pain Syndrome (also known as Reflex Sympathetic Dystrophy, Causalgia, or Sudeck's syndrome)
- o Rheumatoid Arthritis
- o Osteoarthritis
- o Plantar fasciitis
- o Fibromyalgia
- o Hypermobility
- o Back pain
- o Migraine
- o Cluster Headache
- o Multiple Sclerosis
- o Neuralgia
- o Stomach ulcer
- o Endometriosis
- o Irritable Bowel Disease
- o Crohn's Disease
- o Other (Please specify. You can specify more than one thing if you wish) __________________
- o I have not received any diagnosis for my pain condition

You answered that you have received a medical diagnosis of *[diagnosis that was selected]*. Which medical practitioner diagnosed you with this condition (if you recall). Please select ALL that apply.

- o GP
- o Specialist doctor (e.g. rheumatologist or pain specialist)
- o Physiotherapist
- o Occupational therapist
- o Nurse
- o Other (Please specify) ____
- o Don't know/can't remember

Was there an event or injury that triggered the onset of your pain condition(s)?

- Yes
- No

What was the event or injury that triggered the onset of your pain condition(s)?

- Sprain
- Fracture
- Dislocation
- Surgery
- Infection
- Childbirth
- Stressful situation such as bereavement, divorce, or loss of job
- Other (Please specify. You can specify more than one thing if you wish) __________

Neurobehavioral questionnaire

You answered before that you have felt pain in your *[name of painful body part is inserted here]* over the last week. Please answer the following statements on how you feel about your *[name of painful body part is inserted here]*. Select TRUE if the content of the sentence apply to you or FALSE if the content of the sentence does not apply to you.

	True	False
If I don't focus my attention on my [painful limb] it would lie still, like dead weight.	○	○
My [painful limb] feels as though it is not part of the rest of my body.	○	○
I need to focus all of my attention on my [painful limb] to make it move the way I want it to.	○	○
My [painful limb] sometimes moves involuntarily, without my control.	○	○
My [painful limb] feels dead to me.	○	○

Bodily changes

Have you experienced any of the following since the onset of your pain condition? Please only select those things that have **started to bother you** or have **become worse** since the onset of your pain condition. Select ANY that apply.

- Blurred vision
- Needing to change your glasses or contact lens prescription more often
- Peripheral vision loss
- Sensitivity to bright lights
- Hearing loss
- Tinnitus (Ringing in the ears)
- Sensitivity to loud noises
- Losing hair on your head
- Losing hair on parts of your body other than your head *
- Extra hair growth on any part of your body*
- Skin rashes
- Being more susceptible to sunburn
- Changes in the texture of your skin*
- Changes in skin colour*
- Swelling (edema) in any body part*
- Changes in the nails of your hands (e.g. growing faster or slower, or being more brittle) *
- Changes in your toenails (e.g. growing faster or slower, or being more brittle) *
- Allergic reactions on the skin
- Increased susceptibility to illness (for example, becoming more frequently ill, or taking longer to recover from illness)
- Finding your skin takes longer to heal when cut or bruised
- Needing to urinate more often, or finding it difficult from stopping yourself urinate when you ‘need to go’
- Needing to urinate less often, or finding it difficult to urinate
- Loose bowels, diarrhoea, or needing to defecate more often
- Constipation
- Having a “sensitive stomach”
- Nausea
- Increase in weight
- Decrease in weight
- Allergic reactions to food and drink
- Decreased alcohol tolerance
- Increased alcohol tolerance
- Weakness in any part of your body*
- Tremor in any part of your body*
- Problems with balance
- Falling more frequently
- Difficulties walking
- Sweating more*
- Sweating less*
- Dizziness
- Hay fever
- Loss of sexual desire
- Increased sexual desire
- Feeling unusually cold, or finding it difficult to get warm when you are cold
- Feeling unusually hot, or finding it difficult to cool down when you are hot

- One part or specific parts of your body feeling unusually cold*
- One part or specific parts of your body feeling unusually hot*
- Please specify anything else that you have experienced. Even if you have experienced a change to your body or its sensations that you think is odd, unusual, or sounds "a bit crazy", we are interested in hearing about it. You can specify more than one thing if you wish. In the next question you will have an opportunity to explain more about the changes and how they make you feel if you wish. Here, please just list any additional changes if there are any. ____

** These items are the CRPS symptoms that were used for the current study*

Sensory sensitivity

Do any of the following give you pain? Please select ANY that apply.

- Caffeine
- Alcohol
- Bright lights
- Flashing lights
- High-contrast images, such as black and white stripes spaced close together
- Loud or unpleasant noises
- The touch of clothing/water/breeze *
- Particular foods. If yes, please specify ________________
- Particular smells. If yes, please specify _______________
- Cold weather. if yes, then please specify from what temperature your pain starts_________
- Warm or hot weather. If yes, then please specify from what temperature your pain starts __
- Other (Please specify. You can specify more than one thing if you wish)_________________

** This item was one of the CRPS symptoms that were used for the current study*

Patient Health Questionnaire-15 (PHQ-15)

During the past 4 weeks, how much have you been bothered by any of the following problems?

	Not bothered at all	Bothered a little	Bothered a lot
Stomach pain	○	○	○
Back pain	○	○	○
Pain in your arms, legs, or joints (knees, hips, etc.)	○	○	○
Menstrual cramps or other problems with your periods	○	○	○
Headaches	○	○	○
Chest pain	○	○	○
Dizziness	○	○	○
Fainting spells	○	○	○

Feeling your heart pound or race	○	○	○
Shortness of breath	○	○	○
Constipation, loose bowels, or diarrhoea	○	○	○
Nausea, gas, or indigestion	○	○	○
Feeling tired or having low energy	○	○	○
Trouble sleeping	○	○	○
Pain or problems during sexual intercourse	○	○	○

Patient Health Questionnaire (PHQ-9)

Over the last 2 weeks, how often have you been bothered by any of the following problems?

	Not at all	Several days	More than half days	Nearly every day
Little interest or pleasure in doing things	○	○	○	○
Feeling down, depressed, or hopeless	○	○	○	○
Trouble falling/staying asleep, sleeping too much	○	○	○	○
Feeling tired or having little energy	○	○	○	○
Poor appetite or overeating	○	○	○	○
Feeling bad about yourself – or that you are a failure or have let yourself or your family down	○	○	○	○
Trouble concentrating on things, such as reading the newspaper or watching television	○	○	○	○
Moving or speaking so slowly that other people could have noticed	○	○	○	○
Or the opposite – being so fidgety or restless that you have been moving around a lot more than usual	○	○	○	○
Thoughts that you would be better off dead or of hurting yourself in some way	○	○	○	○

Generalized Anxiety Disorder (GAD-7)

Over the last 2 weeks, how often have you been bothered by any of the following problems?

	Not at all	Several days	More than half days	Nearly every day
Feeling nervous, anxious, or on edge	○	○	○	○
Not being able to stop or control worrying	○	○	○	○
Worrying too much about different things	○	○	○	○
Trouble relaxing	○	○	○	○
Being so restless that it's hard to sit still	○	○	○	○
Becoming easily annoyed or irritable	○	○	○	○
Feeling afraid as if something awful might happen	○	○	○	○

Table S1. The number (%) of respondents per group who selected any of the predefined bodily changes that are part of the four categories of the Budapest clinical diagnostic criteria for CRPS (Harden et al., 2010). The sensory item was part of the question: *'Do any of the following make you start to feel pain (when you weren't feeling pain before)?'*, the other items were part of the question: *'Have you experienced any of the following since the onset of your pain condition? Please only select those things that have started to bother you or have become worse since the onset of your pain condition. Select ANY that apply.'* The number (%) of respondents who reported one or more symptoms in each of the four categories, and in at least three out of four categories are depicted. Note that it is not possible to diagnose CRPS based on these questions, because not all symptoms were asked for (e.g. hyperesthesia), symptoms were not specifically related to the affected limb and/or asymmetries between limbs, and signs have not been assessed. This data is depicted to provide some insight in the two groups.

	CRPS (N = 335)	Other limb pain (N = 407)	Chi-square test statistics
**Sensory**			
• The touch of clothing, water, or a breeze	231 (69.0%)	89 (21.9%)	$\chi^2(1) = 166.11$, $p < .001$
**Vasomotor**			
• One part or specific parts of your body feeling unusually cold	207 (61.8%)	119 (29.2%)	$\chi^2(1) = 79.05$, $p < .001$
• One part or specific parts of your body feeling unusually hot	142 (42.4%)	82 (20.1%)	$\chi^2(1) = 43.13$, $p < .001$
• Changes in skin colour	259 (77.3%)	43 (10.6%)	$\chi^2(1) = 339.21$, $p < .001$
≥1 vasomotor symptom	312 (93.1%)	165 (40.5%)	$\chi^2(1) = 221.39$, $p < .001$
**Sudomotor/edema**			
• Sweating more	223 (66.6%)	208 (51.1%)	$\chi^2(1) = 18.04$, $p < .001$
• Sweating less	14 (4.2%)	9 (2.2%)	$\chi^2(1) = 2.37$, $p = .124$
• Swelling (edema) in any body part	277 (82.7%)	142 (34%)	$\chi^2(1) = 170.78$, $p < .001$
≥1 sudomotor/edema symptom	312 (92.1%)	261 (64.1%)	$\chi^2(1) = 87.90$, $p < .001$
**Motor/trophic**			
• Losing hair on parts of your body other than your head	62 (18.5%)	42 (10.3%)	$\chi^2(1) = 10.22$, $p = .001$
• Extra hair growth on any part of your body	115 (34.3%)	60 (14.7%)	$\chi^2(1) = 39.11$, $p < .001$
• Changes in the texture of your skin	214 (63.9%)	112 (27.5%)	$\chi^2(1) = 98.64$, $p < .001$
• Changes in the nails of your hands (e.g. growing faster or slower, or being more brittle)	180 (53.7%)	154 (37.8%)	$\chi^2(1) = 18.75$, $p < .001$
• Changes in your toenails (e.g. growing faster or slower, or being more brittle)	189 (56.4%)	102 (25.1%)	$\chi^2(1) = 75.79$, $p < .001$

• Weakness in any part of your body	285 (85.1%)	316 (77.6%)	$\chi^2(1) = 6.60$, $p = .010$
• Tremor in any part of your body	191 (57.0%)	138 (33.9%)	$\chi^2(1) = 39.66$, $p < .001$
≥1 motor/trophic symptom	328 (97.9%)	359 (88.2%)	$\chi^2(1) = 25.21$, $p < .001$
≥1 symptom(s) in 3/4 categories	308 (91.9%)	154 (37.8%)	$\chi^2(1) = 228.92$, $p < .001$
≥1 symptom(s) in 4/4 categories	207 (61.8%)	51 (12.5%)	$\chi^2(1) = 196.60$, $p < .001$

Abbreviation: CRPS, complex regional pain syndrome.

Table S2. Numbers and percentages of events/injuries that triggered the pain condition. Note that respondents could report multiple events/injuries, thus percentages do not sum to 100.

	CRPS (N = 335)	Other limb pain (N = 407)	Chi square test statistics
None	27 (8.1%)	203 (49.9%)	$\chi^2(1) = 150.23$, $p < .001$
Fracture	114 (34.0%)	24 (5.9%)	$\chi^2(1) = 96.06$, $p < .001$
Surgery	117 (34.9%)	44 (10.8%)	$\chi^2(1) = 62.89$, $p < .001$
Sprain	57 (17.0%)	23 (5.7%)	$\chi^2(1) = 24.67$, $p < .001$
Dislocation	24 (7.2%)	13 (3.2%)	$\chi^2(1) = 6.11$, $p = .013$
Stressful situation	29 (8.7%)	80 (19.7%)	$\chi^2(1) = 17.74$, $p < .001$
Infection	13 (3.9%)	32 (7.9%)	$\chi^2(1) = 5.11$, $p = .024$
Childbirth	7 (2.1%)	18 (4.4%)	$\chi^2(1) = 3.07$, $p = .080$
Other	95 (28.4%)	94 (23.1%)	$\chi^2(1) = 2.68$, $p = .102$

Abbreviation: CRPS, complex regional pain syndrome.

Table S3. Factor loadings on the **first component** of the five-item Neurobehavioral questionnaire by Galer and Jensen (1999), the percentage of explained variance, and Cronbach's alpha, split per group. Items are sorted based on the factor loadings of all respondents.

	All respondents (*N* = 742)	CRPS (*N* = 335)	Other limb pain (*N* = 407)
2. My [painful limb] feels as though it is not part of the rest of my body.	-0.56	-0.53	-0.55
3. I need to focus all of my attention on my [painful limb] to make it move the way I want it to.	-0.50	-0.56	-0.63
1. If I don't focus my attention on my [painful limb] it would lie still, like dead weight.	-0.45	-0.31	-0.28
5. My [painful limb] feels dead to me.	-0.44	-0.49	-0.44
4. My [painful limb] sometimes moves involuntarily, without my control.	-0.23	-0.26	-0.16
Variance explained, %	46%	43%	46%
Cronbach's alpha for item 1, 2, 3 and 5	0.76	0.70	0.77

Abbreviation: CRPS, complex regional pain syndrome.

Table S4. Factor loadings on the **second component** of the five-item Neurobehavioral questionnaire by Galer and Jensen (1999) and the percentage of explained variance, split per group. Items are sorted based on the factor loadings of all respondents.

	All respondents (*N* = 742)	CRPS (*N* = 335)	Other limb pain (*N* = 407)
4. My [painful limb] sometimes moves involuntarily, without my control.	-0.75	-0.68	0.82
3. I need to focus all of my attention on my [painful limb] to make it move the way I want it to.	-0.40	-0.48	0.35
5. My [painful limb] feels dead to me.	0.13	0.36	-0.26
2. My [painful limb] feels as though it is not part of the rest of my body.	0.19	0.40	-0.38
1. If I don't focus my attention on my [painful limb] it would lie still, like dead weight.	0.46	0.18	-0.08
Variance explained, %	21%	23%	22%

Abbreviation: CRPS, complex regional pain syndrome.

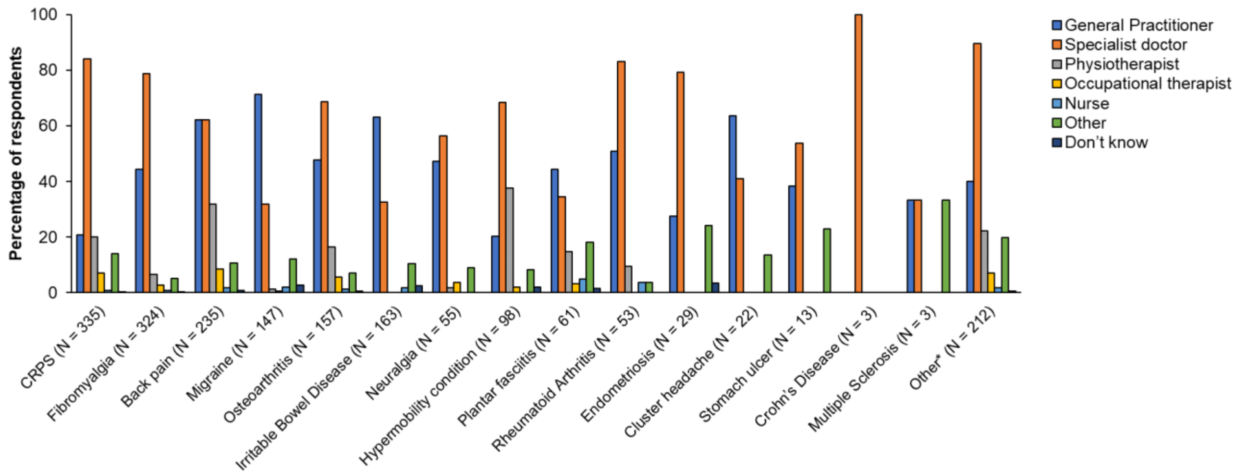

Figure S1. Percentages of medical practitioners that respondents received their medical diagnosis from, split per medical diagnosis. Note that respondents could report multiple practitioners who had provided the medical diagnosis, thus percentages do not sum to 100. CRPS = complex regional pain syndrome.

*'Other' medical diagnoses indicate the number of respondents who received one or more other medical diagnosis.

References

Galer, B. S., & Jensen, M. (1999). Neglect-Like Symptoms in Complex Regional Pain Syndrome. *Journal of Pain and Symptom Management*, *18*(3), 213–217. https://doi.org/10.1016/S0885-3924(99)00076-7

Harden, R., Bruehl, S., Perez, R. S. G. M., Birklein, F., Marinus, J., Maihofner, C., Lubenow, T., Buvanendran, A., Mackey, S., Graciosa, J., Mogilevski, M., Ramsden, C., Chont, M., & Vatine, J.-J. (2010). Validation of proposed diagnostic criteria (the "Budapest Criteria") for Complex Regional Pain Syndrome. *Pain*, *150*(2), 268–274. https://doi.org/10.1016/j.pain.2010.04.030
